# Reduction of Nitrite in Meat Products through the Application of Various Plant-Based Ingredients

**DOI:** 10.3390/antiox9080711

**Published:** 2020-08-05

**Authors:** Karolina Ferysiuk, Karolina M. Wójciak

**Affiliations:** Department of Animal Raw Materials Technology, Faculty of Food Science and Biotechnology, University of Life Sciences in Lublin, Skromna 8 Street, 20-704 Lublin, Poland; karolina.ferysiuk@student.up.edu.pl

**Keywords:** extracts, polyphenols, concentrate, food safety, antioxidants

## Abstract

Nitrite is the most commonly applied curing agent in the meat industry, and is known to affect human health. Nitrites impart a better flavor, taste and aroma; preserve the red-pinkish color of the meat; and prevent the risk of bacterial contamination of the cured meat, especially from *Clostridium botulinum*. Unfortunately, recent research has demonstrated some negative effects of this technique. Certain *N*-nitroso compounds have been shown to stimulate gastric cancer; therefore, most of the research groups are studying the effects of nitrates and nitrites. In this review, we discuss the various food sources of nitrites and nitrates and their current legal requirements for use in meat products. We also discuss the possible changes that might come up in the regulations, the concerns associated with nitrates and nitrites in meat products, and the use of plant-based nitrite and nitrate substitutes. All these topics will be considered with respect to ensuring a high level of microbiological protection, oxidative stability and acceptable sensory quality (color, taste and smell) in meat products.

## 1. Introduction

Nitrite and nitrate are important food additives in cured meat products. These additives have been used since about 3000 BC—when salt naturally contaminated with nitrate was used—through to the 19th and 20th centuries—when curing mechanisms were discovered—up until the present day [1,2]. Despite the advantages of nitrite application in the meat industry (pinkish-red color, and antioxidant and antimicrobial properties), it is harmful to human health. The negative effects of nitrite as a meat additive were first recorded in the early 1950s and 1960s when *N*-nitroso compounds (NOCs) were initially discovered [2].

Previous studies have hypothesized that nitrosamines (and processed meat consumption) are associated with some types of cancer prevalence [3,4]. Subsequently, certain regulations have restricted the amount of sodium nitrite used in the production of meat products. In the European Union (EU), Regulation No. 1333/2008 regulates the use of nitrite [5]. However, in Denmark, less nitrite is added to meat products compared to other countries of the EU, and this practice of reducing nitrite content was accepted by the Commission Decision (EU) 2018/702 [6]. Moreover, according to a report prepared by the Food Chain Evaluation Consortium [7], since 2016, depending on the product type and production process, nitrite has been reduced in various meat products.

Some researchers have focused on the possibility of substituting sodium nitrite with various plant extracts, bacteriocins, selected bacterial strains and high hydrostatic pressure (HHP), in addition to elimination/reduction of nitrite [8,9]. Plant extracts and ingredients appear to be suitable alternatives for nitrite. Plant parts (vegetables, fruits, herbs and spices) contain various types of phenolic compounds that are beneficial to human health. Phenolic compounds demonstrate excellent free radical scavenging activity and therefore might prove to be beneficial [10].

The following review discusses these issues in detail.

## 2. Food Source of Nitrate and Nitrite

Nitrate enters the body mainly through the consumption of vegetables (about 85%), with the remainder entering via drinking water. Nitrite exposure from animal products, such as meat, is small (5%) compared to plant products (80%) [11]. The concentration of nitrate in water increases due to contamination by human- or animal-based natural organic wastes, and due to the intensification of arable agriculture where nitrogen-containing fertilizers are heavily used. The amount of nitrite in water is low because it is oxidized to nitrate—therefore nitrate is a dominant compound in both surface water and groundwater [11,12,13,14]. In general, nitrate and nitrite in drinking water are limited to 50 and 3 mg/L, respectively (as nitrate and nitrite ions). Despite the higher content of nitrate in drinking water, there is no supporting evidence to show that high nitrate content in water is responsible for types of cancer per se [14].

Schullehner et al. [15] conducted a large study of the effect of nitrate content in water on colorectal cancer (CLC). The authors used Danish population-based registers for the time period of 1978 to 2011 and drinking water quality data from a nationwide database. Overall, authors found a link between the level of nitrate in water and cancer (colon cancer, rectal cancer and colorectal cancer). Moreover, the authors noted that the risk of developing CRC increased with increasing nitrate content in water. It was also found that the risk of developing colon cancer was high, even at nitrate levels below the limit for drinking water. This situation led to the conclusion that limits for nitrate in drinking water should be lowered. In another study, Ward et al. [16] collected various data from epidemiological studies of nitrate in drinking water; for example, the authors gathered information on methemoglobinemia and found the risk associated with the incidence of this disease in infants increased with the consumption of water with a nitrate level higher than 50 mg/L. A positive relationship was found between consumption of water from a private well (nitrate <10 mg/L) and levels of methemoglobin in blood. Authors also highlighted a possible link between water with nitrate levels higher than 5 mg/L and thyroid cancer. However, these findings are not obvious; other studies have found no association between nitrate levels in water and cancer [16].

Nitrate is a highly important nutrient for plants. Tamme, Reinik and Roasto [13] noted that nitrate accumulation in plants increases due to environmental stresses. Nitrate content also differs in various parts of the plant: leaf > steam > root. Furthermore, it has been reported that floral parts contain the least amount of nitrite compared to other parts of the plant [11,12]. For humans, vegetables are the main natural source of nitrate. Nitrate levels in vegetables depend on factors such as the type of soil, humidity, storage time, size of the vegetable, plant culture and temperature [12,17]. The major factors that determine the content of nitrate in vegetables, however, are considered to be the intensity of light and the type of applied nitrogen fertilizer. Vegetables from families such as Chenopodiaceae, Brassicaceae, Amarantaceae, Apiaceae and Asteraceae are characterized by the high rate of accumulation of nitrate [17]. The general division of nitrate accumulation in vegetables is presented in Table 1. Spinach, parsley, cabbage and turnip contain a high amount of nitrate; however, these vegetables are usually consumed after cooking, which reduces the nitrate content by about 75% (soups are an exception—the nitrate from plant tissue is transferred to the liquid phase and is increased due to the evaporation of water) [17]. Ranasinghe and Marapana [18] reported that leafy vegetables lose the highest amount of nitrate during cooking compared to other vegetables. Furthermore, the amount of nitrite in vegetables decreases during their storage [11].

The nitrite content of vegetables is also affected by storage conditions. For example, freezing vegetables inhibits nitrate; refrigerated storage has a similar effect, but to a lesser extent. Poor storage conditions result in low nitrate and high nitrite content due to bacterial growth on the vegetable. Low light conditions do not activate the enzyme nitrate reductase and, therefore, the conversion of nitrate to amino acids does not take place. This results in high concentrations of nitrate in plants grown under shady conditions. Furthermore, extreme temperatures can be stressful to plants, in turn potentially slowing the process of photosynthesis or even decreasing the activity of nitrate reductase. Both cases lead to the accumulation of nitrate. Research also shows that storage conditions affect the amount of nitrite in vegetables; for example, storage at room temperature promotes nitrite accumulation [12,17,18]. Lidder and Webb [12] demonstrated that homemade beet juice contains 600 mg/L nitrite after two days of storage at room temperature, as an effect of bacterial nitrate reductases. This process is not observed in the case of pasteurized products (e.g., during industrial production).

**Figure 1:**
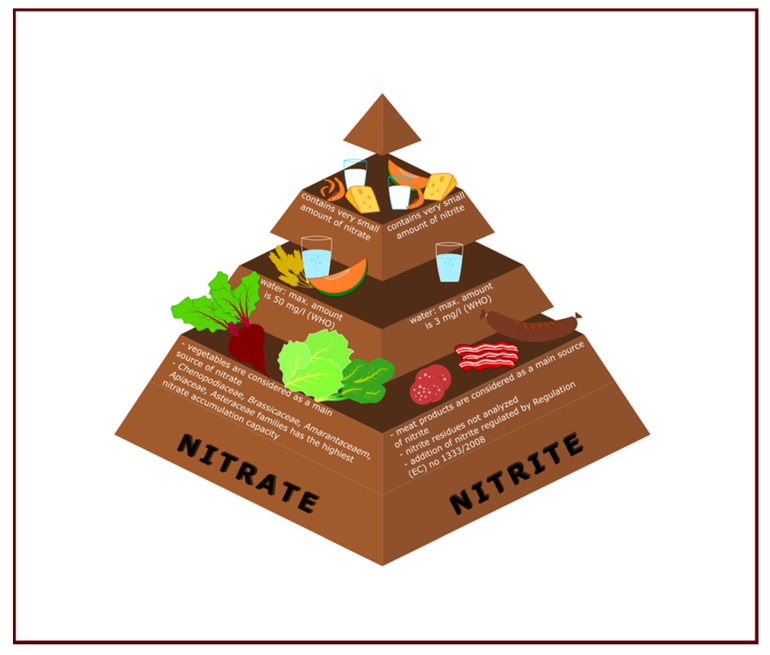
Main sources of nitrite and nitrate in various food’s type.

According to Ranasinghe et al. [18], nitrite content is lower than nitrate content in vegetables. Correira [17] found nitrite levels between 1.1 and 75 mg/kg, and nitrate in a range of 54–2440 mg/kg (kale, turnip, Portuguese cabbage, lettuce, spinach and parsley). In the case of fruits, nitrate content in cantaloupe was in the range of 23–103 mg/kg and nitrite was between 4.6 and 57.5 mg/kg, whereas nitrate content in watermelon and melon was in the range of 13.95–37.7 mg/kg and 16.28–72.5 mg/kg, respectively, and nitrite content in these fruits was in the range of 3.1–8.5 mg/kg and 3.7–16.7 mg/kg, respectively [19]. The amount of nitrite and nitrate in various food sources has been presented in Figure 1.

Cured meat products are another source of nitrite, which is added to stop the growth of bacteria, slow down/stop the process of oxidation, and retain the characteristic pinkish-red color of the meat [9,20]. Nitrate is less effective than nitrite but is applied in the production of fermented meat [21,22]. The amount of added nitrite and nitrate salts depends on the type of product and its current state of knowledge [23].

Merino et al. [24] did not find any effect of boiling on the nitrite content in pork and beef sausage; in contrast, frying reduced nitrite content by about 50%. Twenty-four hours after the addition of nitrite, the authors observed a decrease in the amount of nitrite in all meat products tested (i.e., liver pate, pork/beef sausage, lunch chicken sausage and grilled sausage). Hsu et al. [25] analyzed the nitrite and nitrate content in food and observed that the amount of nitrite present in products cannot be directly translated into the total amount of nitrite added during the manufacturing process. This may be because nitrite is highly reactive. Thus, it can be concluded that vegetables are the primary source of nitrate, and meat products are the primary source of nitrite, in the human diet [25].

Iammarino and Taranto [26] noted that nitrite and nitrate salts should not be added to fresh meat. This conclusion was reached following the examination of 200 samples of fresh meat from various animal sources, in which it was observed that the amount of nitrate ranged from 10.02 to 36.5 mg/kg (nitrate was not detected in chicken meat). They found that equine meat was characterized by a higher level of nitrate than other meats due to the nature of horse feed. Nitrite was not detected in any of the tested samples.

Notably, nitrate is not a neutral substance for animals and can cause their intoxication. As noted by Bolan and Kemp [27], this poisoning is a result of consuming nitrate-rich feed. Moreover, the authors pointed out that ruminants are considered to be more susceptible to intoxication due to the presence of rumen microbes (sheep and horses are less exposed compared to cattle). Nitrate intoxication can result in methemoglobinemia or even progesterone synthesis interference, which could lead to non-infectious abortion. Bolan and Kemp [27] noted that some plant species present greater nitrate accumulation capacity (e.g., maize, oats, barley) than others, e.g., cocksfoot or browntop.

Another study shows that seeds and grains are a poor source of nitrite/nitrate. This research found that the quantities of nitrite and nitrate ions are related to the location of farmland (i.e., industrialized or non-industrialized areas). The authors analyzed the level of nitrite and nitrate in selected cereals and found that nitrite ranged between 0.030 and 0.154 mg/kg, and nitrate from 3.0 to 21.3 mg/kg [28].

The presence of nitrite in dairy products is related to the application of nitrite-containing food additives, or the oral administration of potassium nitrate to dairy cows [29]. According to Zamrik [30], nitrates or nitrites can be detected in milk (because water might be contaminated with these ions), in cheese (due to improper processing during cheese production), or in dairy products generally due to the addition of sodium/potassium nitrite to feed. Sodium or potassium nitrate are typically added to dairy products (e.g., cheese and milk) as antimicrobial agents (e.g., against *Clostridium tyrobutyricum* and *C. butyricum*) and to prevent dairy products from acquiring negative sensory qualities, e.g., cracks, slits and holes (which are caused by the presence of bacteria) [31,32]. This draws attention to the link between the quality of feeding materials and the level of nitrite in dairy products [31]. Nitrate content in dairy products (e.g., Turkish white cheese) was found to vary (from 0.47 to 23.68 mg/kg). The authors speculated that the differences were related to the length of the ripening time and storage conditions—higher levels of nitrate were detected in less ripened products compared to fully ripened products. The same study reported a nitrite level between 0.88 and 1.64 mg/kg [31]. Tudor et al. [32] observed the presence of nitrate, ranging between 1 and 8 mg/kg, in nitrate-free products. They found that the nitrite content was low in all of the dairy products tested (cow and sheep green cheese, and salted matured cow and sheep cheese) (less than 1 mg/kg). A previous study highlighted the presence of nitrate in products manufactured in the small-scale dairy industry, mainly due to hygiene deficiencies [31,32]. Genualdi et al. [33] noted that nitrite is not a usual additive in the making of cheese; its presence can result from the reduction of nitrate during the ripening process or xanthine oxidase in milk.

Similar to dairy products, seafood also contains very low amounts of nitrite or nitrate. Notably, nitrites and nitrates are toxic to fish. Chiesa et al. [34] examined fish, shrimps and bivalves from various processing and environmental settings. They detected nitrite in all samples; however, the presence of nitrate varied from 2.8 (mussels) to 89.3 µg/kg (clams). The authors suggested that these results were due to the growing system and location. In addition, breeding and preservation programs affect the levels of nitrates in fish, whereby animals from farms have significantly higher nitrate levels than wild fish. Similar results were obtained for smoked fish in comparison to fresh and frozen fish [34].

## 3. Problems Associated with Nitrates and Nitrites in Meat Products

As mentioned earlier, both nitrite and nitrate can be found in various food products and in various amounts. A key consideration is their impact on human health. To address this issue, we first discuss what happens to these chemicals following consumption.

In the acidic environment of the stomach, nitrate is transformed into nitric oxide (NO) and other metabolites. Next, in the upper part of the small intestine, nitrate is absorbed into the blood and finally secreted by the salivary glands [13,35]. The amount of nitrate in the saliva is 10 times higher than that in the blood, because nitrate is excreted by the salivary glands. Moreover, the bacteria present in the mouth, particularly on the tongue (e.g., *Veillonella* species, *Staphylococcus epidermidis*, *Actinomyces* species, *Rothia* species), reduces about 20–25% of the nitrate to nitrite (which is about 5% of the total ingested nitrate) [12,13,35,36]. However, scientists have observed that, in general, around 70–80% of total nitrite exposure is due to the reduction of nitrate by bacteria, and that ingested nitrate is the source for nitrite conversion [13].

In the human digestive system, therefore, nitrate is converted to nitrite. Thus, it may be considered that vegetables, which are rich in nitrates, pose a threat to human health. However, vegetables contain various type of antioxidant compounds, which suppress the formation of harmful chemicals, i.e., nitrosamines [17]. The oxidation-reduction property of antioxidants such as ascorbic acid and alpha-tocopherol helps in the reduction of nitrosating agents to NO [12,36]. Thus, the exogenous nitrate present in the human body comes mainly from a nitrate-rich diet. However, the primary source of nitrite in the human body is not vegetables, but cured meat products [35].

L-arginine is the main source of NO, which is endogenously formed in the human body. Nitric oxide synthases (NOS) from the amino acid and NO synthetase in human cells is the typical pathway for this molecule [13,35,37]. Furthermore, the amount of NO formed from exogenous nitrate is about 10,000 times higher than that of NO formed through the endogenous path [37]. As mentioned earlier, nitrate is usually absorbed from the small intestine; however, the kidneys salvage some of the nitrates via selective reabsorption. It is notable that about 80% of the nitrate is excreted from the body in urine [37]. Bryan and Grinsven [36] pointed out that, depending on the metabolism, nitrite shows both positive and negative effects—some pathways can lead to the formation of NO, whereas others lead to the formation of *N*-nitrosamines. Nitrate shows bactericidal effects in the stomach—when swallowed in saliva, nitrate comes in contact with the acidic environment of the stomach and is converted to nitrous acid, which is then reduced to NO and other reactive nitrogen species [12,38]. NO is also an important chemical participating in immune reactions, the regulation of cardiovascular homeostasis, and vasodilation [39]. Is also responsible for the control of regional blood flow and blood pressure. Moreover, it acts as a neurotransmitter in both peripheral and central nervous systems (e.g., in the regulation of gastric emptying and long-term potentiation). Furthermore, due to the presence of the peroxynitrite radical (ONOO^−^), which is formed from the excessive NO present, organisms are protected from various pathogens such as parasites, bacteria and fungi [37]. However, nitrate consumption also has negative effects—this topic was discussed earlier.

Nitrite is far more reactive than nitrate, and is more reactive in an acidic environment such as that of the stomach [36]. Nitrous acid is a nitrosating agent. It takes part in the reactions where endogenous NOCs are formed. However, NO can also be a source of the formation of nitrites and nitrates, which can circulate in the human body [2,14,16]. High levels of nitrite in the human body can lead to diseases such as methemoglobinemia, which is a life-threatening condition. This condition occurs when the oxygen-carrying capacity of blood is diminished due to the presence of methemoglobin [16]. Infants under the age of six months are sensitive to this disease, which results in the formation of an immature methemoglobin reductase system and the production of a small amount of stomach acid [16,36]. Another negative effect associated with nitrites is the formation of N-nitrosamines. N-nitrosamines can be formed in meat during the production process, during preparation at home, and in the gastrointestinal tract after consumption [7]. They are formed from nitrite, secondary amines and nitrosating agents [36,40]. In general, N-nitrosamines can be divided into two groups: nonvolatile (NVNA) (e.g., N-nitrosoproline (NPRO), N-nitrososarcosine (NSAR) and *N*-nitrosomethylaniline (NMA)) and volatile (VNA) (e.g., N-nitrosodimethylamine (NDMA), N-nitrosomethylethylamine (NMEA) and *N*-nitrosodiethylamine (NDEA)) [41,42]. Most volatile nitrosamines are classified as group 2B—possibly carcinogenic to humans [42]. The quantity of nitrosamines present in processed meat products depends on their type. According to Hermann, Grandby and Duedahl-Olesen [41], the level of nitrosamines in meat products may be below the detection limit (<1 µg/kg). N-Nitrosamines (NAs) are carcinogenic in nature. As Bryan and van Grinsven [36] explained, most N-nitrosamines are organ-specific, which means that some have a carcinogenic effect in selected organs. Moreover, they also present teratogenic effects [13]. At present, it is hypothesized that there is a positive association between the level of nitrite addition and the formation of *N*-nitrosamines, but the association is not linear [25,29]. Moreover, because cooking at high temperatures and the processing of cured meat also lead to the formation of NOCs, unabsorbed nitrosylated heme from red meat in the colon can be a source of NOCs. In the presence of heme, amides and amines can be nitrosated [43].

An experiment on mice fed with sodium nitrite (0.5%) and butylurea (0.85%) showed the increased formation of malignant lymphomas. Furthermore, the combination of nitrite and secondary amines contributed to the formation of lung adenomas in mice. It was reported that, consistent with an increase in the amount of sodium nitrite (with the amount of piperazine remaining constant), there was an increase in lung adenomas; however, other experiments have shown no effect of sodium nitrite on the formation of tumors [3].

## 4. Legislation—Now and Future Goals

As mentioned earlier, the addition of nitrite has both positive and negative effects. Because the primary factor responsible for the characteristic features of cured meat products is nitrite, which has higher activity than nitrate, it was deemed necessary to regulate its use.

The first regulation associated with the addition of nitrite to meat was created in 1934 in Germany. The nitrite curing salt law (Nitri–Pökelsaltz–Gesetz) allowed nitrite to be applied (in amounts no higher than 0.6% and no lower than 0.5%) in a mixture with salt. Much later, in the 1950s, the regulation was made more specific to raw ham: sodium nitrite was permitted to be added at a rate of 150 mg/kg into raw ham and 100 mg/kg into ready-to-eat meat products [40]. These guidelines were applied to the Directive No. 95/2/EC from 1995, where the indicative amount of sodium nitrite added was set at 150 mg/kg for canned meat products and other cured meat products [40,44]. However, in 2006, the limit to nitrite addition was again changed, with the maximum amount permitted to be added during the manufacture of sterilized meat products set at 100 mg/kg; for some traditional products (e.g., Dunajská klobása and similar) the limit was set to 180 mg/kg. [23]. Among the European countries, Denmark is an exception. Levels of the additives E249–E250 are much lower in Danish legislation compared to that of the European Union (EU) (according to the Danish legislation, 60 mg/kg NaNO_2_ can be added to most products) [45]. Furthermore, according to the Commission Decision (EU) 2018/702 [6], the use of the additive E250 in quantities significantly lower than those provided for in the EU law was authorized.

According to Regulation (EC) No 1333/2008 of the European Parliament and of the Council, the maximum amount of nitrite that can be added to sterilized meat products is 100 mg/kg; for other meat products, the limit is 150 mg/kg [5]. However, there are exceptions to this rule; for example, for some traditionally cured meat products, the maximum amount of nitrite allowed is 175 mg/kg (Entremeada, entrecosto, chispe, orelheira e cabeca, toucinho fumado and similar; and rohschinken, trockengepökelt and similar), 50 mg/kg (tongue, cured) or 180 mg/kg (vysočina, selský salám, turistický trvanlivý salám and similar). These are not the only differences from the regulation of 2006. In this regulation, the maximum residual levels of nitrite salt were examined and included in the document; the regulations from 2008 did not contain this information [5,6].

In contrast, according to United States of America legislation—the Federal Regulations [46]—depending on the application and product type, in general, levels of sodium nitrite should not exceed 200 ppm in the finished product; for sodium nitrate, this should be no more than 500 ppm. In the Canadian Food and Drug Regulations [47], the total amount of nitrites (sodium nitrite added alone or on combination with potassium nitrite) should not exceed 200 ppm for most products. For curing side bacon, the total amount should not exceed 120 ppm. In the Canadian legislation, the quantity of nitrites and nitrates is calculated at the input level. In the Australia New Zealand Food Standards Code (Standard 1.3.1—Food Additives), the maximum permitted levels of nitrites (potassium and sodium salts) are expressed as the total amount of nitrites and nitrates, calculated as sodium nitrite [48]; for example, in contrast to EU legislation, Standard 1.3.1. allows the application of 50 mg/kg of nitrite salts to commercially sterile canned cured meat; for dried meat, cured meat, slow dried meat and processed comminuted meat, poultry and game products, the maximum permitted level is 125 mg/kg (for nitrates—500 mg/kg) [5].

E249 and E250 are important additives in the meat industry. However, under certain conditions, they act as nitrosating agents, thereby helping in the formation of *N*-nitrosamines. According to the Food Chain Evaluation Consortium (FChEC) report [7], it is possible to reduce the use of nitrite in meat products, but this requires some additional steps in their preparation.

## 5. Beneficial Effects of Plant Extracts in Meat Products

The application of new food additives, flavorings and enzymes is regulated by Regulation (EC) 1331/2008 of the European Parliament and of the Council [49]. After receipt of an application, the European Commission initiates a procedure. The Commission also requests the opinion of the European Food Safety Authority (EFSA) [49]. More detailed information about applications is regulated by Commission Regulation (EU) 234/2011 [50] This regulation clearly indicates that use of food enzymes and additives should be technologically justified. According to 234/2011, applications must be drafted according to the model presented in the Annex. In general, applications must contain: a letter, a technical dossier (all administrative data, and all data requested for the risk assessment and risk management) and a summary of the dossier. Toxicological tests must be performed according to Directive 2004/10EC (if tests are performed in EU) or the OECD Principles of Good Laboratory Practice (GLP) (OECD, 1998) (outside the EU). In a detailed manner, Commission Regulation 234/2011 [50] specifies the kind of information that should be presented in the applications; for this reason, the issue will not be widely discussed, and only some issues will be mentioned. Information that must be included in the application includes the identity of the food additive; its stability, reaction and impact in the foods in which it is used; and biological and toxicological data. The Regulations also specify that specific, separate data is required for risk assessment of food enzymes, additives and flavorings **[50]**. On the official website of the European Union, the practical guide for the authorization procedure can be found [51]. Furthermore, point A.09 of the Summary Report of the Standing Committee on Plants, Animals, Food and Feed held in Brussels on 17 September 2018 [52] contains information related to plant extracts. Depending on their purpose (substance of flavoring properties or substance of technological function) their status can be considered as flavoring or food additive (in which case, food additive legislation shall apply). To provide more clarity, in 2019 EFSA created a “Best Practice Guidance Document” concerning plant extracts and their legislation status. The guide was created to eliminate all ambiguities related to the legal status of extracts resulting from their traditional use as a flavoring agent. According to the guide, to determine the correct regulatory status of plant extracts, the intended application in the final food and extraction process should be considered [53].

In the case of extracts, Directive 2009/32/EC [54] states that extraction solvents cannot contain toxicologically dangerous substances and should satisfy the specific criteria of purity. Moreover, the Directive also lists extraction solvents which are permitted for use in extraction processes (ethanol, acetone, butyl acetate, etc.) and solvents for which some specific conditions should be applied (hexane, dichloromethane, etc.). Depending on the target substances to be extracted (polyphenols, anthocyanins), it is possible to use various solvents for maximum efficiency, e.g., an aqueous mixture of methanol, ethanol and acetone; only methanol; only ethanol; or their aqueous solutions [55].

As noted by Oroian and Escriche [55], ethanol is the most frequently used solvent for extraction. Although methanol is cheaper, ethanol is nontoxic and has GRAS status, which means that ethanol can be used safely in food. In the case of methanol or other solvents, their residues must be removed from the extract. For example, the maximum residue limit in extracted foodstuffs or food ingredients for methanol is 10 mg/kg, and for methyl acetate is 20 mg/kg (in coffee or tea); in the preparation of flavorings from natural materials, the maximum residue limits in foodstuffs resulting from the use of extraction solvents are 1 mg/kg for methyl acetate, 2 mg/kg for diethyl ether and 0.02 mg/kg for dichloromethane [54]. Oroian and Escriche [55] also highlighted techniques for extract purification and isolation (e.g., ultrafiltration or macroporous adsorption resins). It should be noted that other extraction processes are available, such as subcritical water extraction, pulsed electric field, ultrasonics, microwave-assisted extraction, supercritical fluid extraction, solid phase extraction and pressurized liquid extraction [55,56]. Each of these methods has advantages and disadvantages; however, the purpose of this article is not a detailed description of extraction techniques, and hence they are mentioned only.

It is noteworthy, however, that the efficiency of extracts not only depends on the solvent and extraction techniques, but also on the raw material. For example, Sulas et al. [57] analyzed the antioxidant capacity of *Chrysanthemum coronarium* L (garland) in two different phenological stages—vegetative and flowering. The extract from the herb at the flowering stage is characterized by a higher antioxidant capacity. Moreover, the authors also found differences between plant organs: stems and heads showed lower antioxidant capacities than leaves. Elwekeel, Elfishway and AbouZid [58] also noted differences in the content of silymarin depending on the maturity stage of milk thistle (*Silybum marianum*) fruits; the highest silymarin amount was found in fully mature fruits [58]. Differences in antioxidant ability and polyphenol composition were also found between herbs belonging to the *Epilobium* genus (Onagraceae). Authors found that *E. pariviflorum* presented the highest antioxidant capacity from all five examined species, and *E. angustfolium* was characterized by the most varied and richest composition of the selected herbs [59]. Ghasemi et al. [60] noted that the climatic and geographical conditions of regions of Iran influenced the flavonoid and phenol contents and antioxidant activity of green husk extracts from walnut (*Juglans regia* L).

In addition to extracts, the product itself should also be considered. Various meat product types exist: hams, cooked sausages, fresh sausages, fermented sausages, air-dried meat products, liver pâté, patties, nuggets, etc. Each of these has its own specifications and characteristics which translate into safety, shelf life and general quality. For example, cooked sausages, due to their high water activity, are perishable; in contrast, cured and air-dried products are considered to be more stable due to their production process (drying). The recommended storage temperature for nuggets is around −20 °C, and for spreadable liver sausage is between 0 and 2 °C. In addition, the composition of individual meat products is different; these can be produced from various meat types and cuts, and various spice mixtures can also be applied [61]. All of these factors should be taken into account when experimenting with possible measures to reduce nitrate. 

Before proceeding to the next section, the authors would like to draw attention to one more issue—consumer acceptance. The previous sections describe the purpose of nitrate and nitrite applications, and the health aspects associated with their use. Based on the above outline, reducing nitrate in meat products would appear to be the correct approach. However, this should be associated with the consumer’s desire to buy such a product. An alternative for nitrite could be identified and new meat products could be created, but it remains to be seen whether this would be sufficient for consumers to consider making a purchase. According to Hung, de Kok and Verbeke [62], consumers are interested in meat products with lower nitrite/nitrate amounts, finding them to be “more natural”. However, the authors also noted that decisions about the purchase of new meat products are associated with an awareness of the purpose and function of nitrate/nitrite, their reason for application, and their consequences [62]. Thus, a successful reduction of added nitrite in meat products, together with plant fortification, would result in a number of benefits to customers, such as a decrease in cancerogenic compounds in products, in addition to an increase in bioactive compounds.

Furthermore, the cost of plant additives should be considered. Compared to other methods of preparation, freeze drying appears to be more expensive than spray drying or convective drying. However, previous research has noted that the final costs of each method comprise various components costs (e.g., in the case of freeze drying, sublimation of ice, freezing, bound water elimination and total energy consumption) [63]. Cost considerations pose another challenge to the reduction of nitrite in meat.

### 5.1. Color

One of the most significant benefits of nitrite in the preparation of meat products is that the meat retains its color. The pinkish-red color is formed through a series of complicated steps, and is due to the formation of nitrosylmyoglobin (NO-myoglobin). Nitrite itself is not responsible for the color of the meat. NO-myoglobin is formed by the binding of myoglobin (Fe^2+^) with NO (formed previously from N_2_O_3_). Under heat treatment, unstable NO-myoglobin degrades to form nitroso-myochromogen, which is a stable, red protein. However, under the influence of ultraviolet (UV) light and in the presence of harmful bacteria, the characteristic color is lost [64,65]. Table 2 presents plant extracts and their effects on meat products. Green leafy vegetables are a good source of nitrate; therefore, they can be used as an alternative to synthetic nitrite [66]. For example, parsley (*Petroselinum crispum* Mill.) and spinach (*Spinacia oleracea* L) contain high quantities of nitrate (average content: 1000–2500 mg/kg) [11]. As mentioned previously, redness of meat products depends on NO, which is a source of nitrite. Riel et al. [67] did not identify any differences in a lightness (L*) parameter for mortadella-type sausage with various amounts of parsley extract powder (PEP) added, but differences were found for a redness (a*) parameter. These authors observed that the redness in the product faded, but the percentage reduction depended on the amount of PEP applied (a reduction of about 37.29% for samples with 1.07 g/kg of PEP addition, 12.61% for 2.12 g/kg of PEP addition, and 6.56% for 4.29 g/kg of PEP addition, compared to 2.20% in the sample with a 1.8% nitrite addition). An increase in the value of a yellowness (b*) parameter was observed with an increasing amount of PEP added, which was not reported for spinach extract. The authors concluded that the increase in yellowness (b*) was due to the presence of plant pigments [67,68]. Horsch et al. [68] noted that the addition of celery (either in the form of plain concentrate or concentrate with citric acid supplemented) did not affect the redness of the product; the a* value was similar to that of the samples with the addition of sodium nitrite. However, they recorded changes in the lightness and yellowness parameters with an increasing amount of celery concentrate—pork ham was found to be more yellow and darker than the control sample. In another study, the authors compared the effect of young radish and commercial vegetable powder (250 g/kg) on various parameters of cooked pork sausage. According to their results, the powders created darker and less red products than sausage with the addition of sodium nitrite. However, the redness value (a*) for samples with young radish added was greater than that of the product with a vegetable powder addition [69]. Khaleghi et al. [70] observed that barberry extract decreased the lightness value (L*) of beef sausages compared to a control sample to which 120 mg/kg of nitrite was added. The authors also pointed out that the combination of 90 mg/kg plant extract with nitrite not only negatively affected the quality of the meat product by increasing the formation of thiobarbituric acid reactive substances (TBARS), but also decreased the redness of the product over time. The addition of red wine (7.5%) to chouriço (dry-cured sausage) did not change the value of the redness parameter (a*), which recorded the same high value as achieved with the addition of sodium nitrite (150 ppm). The authors also observed that red wine (7.5%) in combination with garlic (1%) increased the yellowness (b*) of the product, which was thought to occur because of the interaction between the two ingredients [71]. Similar results were obtained for beef sausages treated with grape pomace (1% and 2%) combined with nitrite (30 mg/kg). In this case, grape pomace lowered the values of the redness (a*) and lightness (L*) parameters of beef sausages (in comparison to the control sample with the addition of 120 mg/kg nitrite) [72]. Moawad et al. [73] reported that nitrite (100 or 125 mg/kg) in combination with green tea catechins (300 mg/kg) protects raw cured sausage from pigment deterioration. They recorded the highest values for the b* parameter after the addition of catechins. Aliyari et al. [74] reported that samples (beef sausage) with no added nitrite but with the addition of the highest quantities of extracts of pomegranate peel (*Malase torshe saveh* variety) and pistachio green hulls (*Ahmad aghaei* variety) (1250 ppm) exhibited more yellowness (b*) than the control sample with nitrite addition (120 ppm). The lightness (L*) value of beef sausages increased with an increasing amount of pistachio green hull extracts (from 250 to 1250 ppm with the simultaneous reduction of nitrite from 100 to 0 ppm). However, the value of the a* parameter decreased in almost all of the treated samples. This approach could also be applied to plant wastes (e.g., fruit peel). Manihuruka et al. [75] suggested the use of red dragon fruit (*Hylocereus polyrhizus*) peel as a natural colorant and antioxidant. Although the peel contains betacyanins, the redness of beef sausage was found to be less intense, which could be due to the degradation of betacyanins after the boiling process (60–65 °C, 60 min.). They also found an increase in the value of yellowness (b*) with increasing quantities of dragon fruit peel extract added in beef sausages, which they attributed to the presence of betaxanthin. The addition of 0.2% of adzuki bean extract in the preparation of uncured pork sausage was found to increase the redness of the product, with a simultaneous reduction in the lightness value [76]. Similar results were obtained for pork sausage prepared with various proportions of annatto seed powder (0.025%, 0.05%, 0.1% and 0.2%). Another study showed that, with increasing amounts of *Bixa orellana* (L) seed powder, the values of a* and b* parameters increased and the L* value decreased. It is notable that nitrite levels were reduced in the sample to 37.5 ppm in comparison to the control sample (150 ppm) [77]. Çemtekin et al. [78] reported that there were no significant differences for the L* parameter when they tested samples (turkey meat model system) following the addition of *Viburnum opulus* (L) and *Crataegus monogyna*, and after 30 days of storage under aerobic and anaerobic conditions. They obtained differences in values of the a* parameter, which increased with an increasing quantity of *V. opulus*. Lin et al. [79] tested the effects of different concentrations of green tea extract (GTE; 0.02%, 0.05%, 0.1%, 0.2% and 0.5%) in pepperoni, and obtained the highest values of color (L*, a*, b*) parameters for salami treated with 0.05% GTE in combination with 0.009% nitrite. Vossen et al. [80] tested the effect of dog rose (*Rosa Canina* L.) fruit extract on the color parameters of frankfurters. At the end of the tested storage period, there were slight differences observed in the values of the color parameters; the value of yellowness had increased and the addition of a greater quantity of the extract increased the value of the a* parameter. Hayes, Canonico, and Allen [81] studied the effect of tomato pulp powder in luncheon roll products, and found that the values of redness (a*) and yellowness (b*) increased and the value of lightness (L*) decreased. Another group of researchers studied the effects of grape seed extract with olive pomace hydroxytyrosol, and chestnut extract with olive pomace hydroxytyrosol, on the color values of Cinta Senese dry-fermented sausage. The values of redness were increased by the addition of grape seed extract, which the authors concluded was probably an effect of the formation of Zn-protoporphyrin [82]. Shin et al. [83] reported that 2% preconverted nitrite from Swiss chard powder positively affected the formation of nitrosoheme pigments in cooked pork patties. Swiss chard powder was also tested in combination with 60 ppm of nitrite, which resulted in satisfactory results for the a* parameter obtained. Sucu and Turp [84] observed an increase in the values of the redness parameter in sucuk with the addition of beet (*Beta vulgaris*) extract. They obtained the highest values from the sample containing 0.35% of the beet extract. They also noted that beet extract decreased the value of yellowness (b*) and increased the value of lightness (L*) during the first few days of storage. Jin et al. [85] studied the effects of various plant extracts and powders, and recorded a positive effect on the CIE L*a*b* parameters in pork sausage; the predominant effect was an increase in the values of the redness (a*) parameter. Fruits are not rich in nitrates [11], and due to a lack of information about nitrite substitution using plant extracts, scientists have investigated extracts from karanda (*Carissa carandas* Linn), sour cherry (*Prunus cerasus* L.) and blackcurrant (*Ribes nigrum* L.) leaves [86,87]. Karanda is a herb from Thailand that is used in folk medicine to treat intermittent fever, earache and oral inflammation. The dark violet fruits of this plant are rich in anthocyanins and contain high amounts of vitamin C. However, anthocyanins do not improve the color of meat products. Researchers have studied the effect of anthocyanins after 36 h of fermentation of sausages, and found that the color of the fermented sausages had higher values of yellowness (b*) and lightness (L*) parameters compared to the control sample with nitrite addition [86]. Nowak et al. obtained similar results [87]. They observed that *P. cerasus* and *R*. *nigrum* did not increase the value of the redness (a*) parameter of pork sausage, and that the extracts were responsible for the high value of the yellowness (b*) parameter. Researchers are also evaluating the possibility of using vacuum-packed extracts to preserve the qualities of meat products. Marazzeq et al. [88] reported that it is not possible to completely replace nitrite with olive leaf extract (OLE). They conducted various trials with different combinations of OLE and nitrite, and found that with decreasing levels of synthetic additives, there was a decrease in the redness parameter. The sample with total nitrite replacement demonstrated the lowest value of the redness parameter after 12 weeks of storage. According to Nowak et al. [87], with the application of plant extract, it is necessary to include a natural color component during the preparation of the meat product.

### 5.2. Antioxidant Properties

The method of protective effect imparted by nitrite against the oxidation process in cured meat products is similar to that of protecting the discoloration of the meat product. In meat products, free ferric ions can act as a catalyst of oxidation. After curing, NO binds to iron from heme and inhibits the process of lipid peroxidation, thereby enhancing product stability during storage [64,65]. In addition, S-nitrosocysteine is formed due to the utilization of oxygen during the oxidation of NO to NO_2_ [40,64]. Plant extracts demonstrate strong antioxidant activities [89]. Polyphenols can inhibit the propagation of free radicals and their formation through iron chelation [66]. Table 3 shows the antioxidant effects of various plant extracts on meat products.

Sueprasarn et al. [86] conducted studies on the effect of karanda extract (0.05% *w/w* concentration) on the storage of Nham sausage. According to their results, karanda extract did not inhibit the process of lipid oxidation during 30 days of storage, and its activity was similar to nitrite. There was a significant increase in the value of TBARS in fermented sausage, which could be a result of the degeneration of antioxidant chemicals through light and heat exposure. Nowak et al. [87] used extracts from sour cherry and blackcurrant leaves, and found that the reduction of malondialdehyde (MDA), after 14 and 28 days of storage, was similar to that of nitrite. The leaves of *R. nigrum* show a stronger antioxidant activity than those of *P. cerasus*, which might be attributed to the higher amounts of polyphenolic compounds present in the leaves. Kim et al. [66] demonstrated that the amount of plant extract applied is very important in inhibiting the formation of TBARS in meat products. In their experiments, they recorded lower values of MDA in samples with the addition of the highest quantity of fermented spinach extract, compared to the values of the control sample. Kurćubić et al. [89] also showed that the concentration of the plant extract is an important factor in maintaining the quality of the meat product. They conducted experiments with two samples using extracts of *Kitaibelia vitifolia* of 30.0 (concentration 3% *w/v*) and 12.5 (concentration 10% *w/v*) g per kg of meat dough. According to their results, the application of 10% concentrate at a lower dose provided greater protection than samples with 3% concentrate applied at a higher dose. In general, *K. vitifolia* extract showed greater antioxidant activity than nitrite. Another study showed that higher quantities of barberry extract resulted in better antioxidant activity during the first few days of storage, but this effect decreased over time. The authors suggested that there was an antagonistic effect between phenolic compounds of barberry extract and nitrite [70]. A similar observation has been reported with regard to TBARS—two compounds at higher quantities work in an antagonistic way [70]. Sucu and Turp [84] observed similar results in sucuk after testing the extract of beet. They observed that although *B. vulgaris* contains polyphenolic compounds, they did not decrease the TBARS values during storage. Riazi et al. [72] reported that higher quantities of natural antioxidants do not lead to a reduction in the formation of MDA. However, Moawad, Abozeid and Nadir [73] reported that 300 mg/kg of green tea catechins in combination with 100 or 125 mg of nitrite added to dry-fermented sausage shows stronger antioxidant properties than those of control samples. Lin et al. [79] reported that although the addition of GTE (0.05%) to salami contributes to the lowering of TBARS values, its combination with nitrite (0.009%) is not effective. As previously suggested, the combination of both plant extract and nitrite might result in a pro-oxidant status. For example, Hayes et al. [81] reported a pro-oxidant effect of nitrite and tomato pulp powder in luncheon roll. Jin et al. [85] also recorded an increase in the value of TBARS in samples prepared with various plant extracts. However, Shin et al. [83] reported the synergistic effects of nitrite and plant extract against lipid oxidation in pork patties. Ko, Park and Yoon [69] demonstrated that vegetable powders (young radish and a combination of cabbage and Chinese cabbage) can prevent lipid oxidation in cooked pork sausages during storage of up to 20 days. Moarefian et al. [90] reported that after 30 days of storage, all sausages treated with various quantities of the essential oil (EO) of *Mentha piperita* combined with nitrite provided satisfactory results. According to their results, 20 ppm EO and 100 ppm of nitrite added to the product demonstrated the strongest lipid oxidation inhibition activities, compared to 120 ppm of NaNO_2_ alone. Adzuki bean (*Vigna angularis*) is a rich source of catechins, anthocyanins and chlorogenic acid. Jayawardana et al. [76] studied samples prepared with Adzuki bean extract (0.2%, 0.3%, 0.4% and 0.5%) and found that the extract showed a good inhibitory effect of lipid oxidation, which was similar to that of 0.1% BTH. The authors concluded that 0.2% of the extract was optimal to inhibit the lipid oxidation process in comparison to the product prepared with 0.008% sodium nitrite alone. Çemtekin et al. [78] noted that guelder rose (*V. opulus* L) and hawthorn (*C. monogyna*) decreased the amount of MDA formed in cooked turkey during the first 30 days of storage under aerobic and anaerobic conditions. The authors noted that the highest quantities of the plant extracts showed the greatest inhibitory effect toward the formation of TBARS. Similar observations were recorded by van Cuong and Thoa [77]. They added the seed powder of *B. orellana* (L) to the product, which contributed to the reduction of primary and secondary lipid oxidation products to an extent similar to that achieved in the control sample (150 ppm nitrite) [77]. Another study demonstrated that the antioxidant capacity of the product increases with an increasing quantity of plant extract. For example, *H. polyrhizus* peel extract added to the product increased antioxidant activity from 49.71% (control sample) to 54.76%, 60.89% and 72.94%, respectively, with 20%, 30% and 40% of the extract. Similar results were obtained for the TBARS parameter [75]. Another study reported the use of extracts of pomegranate peels and pistachio green hulls to decrease the formation of MDA [74]. Sharma et al. [91] found the addition of 5000 ppm of turmeric powder to be the most effective for slowing down the process of lipid oxidation.

### 5.3. Antimicrobial and Antifungal Properties

Nitrite also shows strong microbicidal activity against some foodborne pathogens, such as *Clostridium botulinum*, *Listeria monocytogenes* and *Escherichia coli*. The bacteriostatic property of nitrite in cured meat products is associated with the formation of NO and/or HNO_2_. Researchers hypothesize that the bactericidal activity results from the inactivation of iron–sulfur proteins of the bacteria by the presence of NO in the meat product. The bactericidal activity of nitrite is affected by various factors, such as salt concentration, heat treatment, various curing ingredients and pH value [64,65]. Plant extracts contain various compounds (e.g., phenolics, flavonoids, tannins and saponins) that show strong antimicrobial activity, which is related to the presence of the hydroxyl group (-OH). The mechanism of action of phenolic compounds is based on disrupting the structure of the proteins present on the bacterial cell wall. This results in the leakage of the cellular components which kills the bacteria [92]. Table 4 presents data on plant extracts and their effects on meat products. Riel et al. [67] added parsley extracts to mortadella-type sausage. According to their results, with an increase in the amount of the extract, the bacterial cell count of *L. monocytogenes* decreased. The researchers concluded that a higher amount of vegetable extract causes higher nitrate and, therefore, nitrite formulation; examination proved that the greatest inhibition properties were in samples with the highest extract addition (4.29 g/sausage meat). Ko, Park and Yoon [69] suggest the possibility of applying vegetable powders (especially from young radish) as a natural source of nitrite for microorganism inhibition.

Furthermore, celery concentrate (75.6 g) inhibited the growth of *L. monocytogenes* in a manner similar to that achieved by 100 mg/kg of sodium nitrite. Similarly, pork ham treated with 151.2 g of celery concentrate inhibited the growth of *L. monocytogenes* in a manner similar to achieved by 200 mg/kg sodium nitrite. However, the effect of celery concentrate was most effective in combination with 10% citric acid [68]. Xi et al. [93] observed that the addition of natural powders or extracts to pork sausage (meat model system) resulted in the inhibition of growth of *L. monocytogenes.* Cranberry powder, GTE, grape seed extract and cherry, lemon and lime powders (in higher quantities) reduced the bacterial count in the product. Bakhtiary et al. [94] reported that EOs in combination with sodium nitrite (100 or 200 mg/kg) synergistically inhibited the growth of *Clostridium sporogenes* and *C. perfringens*. The authors reported that EOs from *Satureja bachtiarica Bunge* showed the strongest inhibitory activity in beef fillets inoculated with *Clostridium* species during storage of up to 30 days. The antimicrobial effect of EOs is mainly associated with their chemical composition [94]. Another study analyzed the counts of *E. coli*, *Salmonella* sp. and *S. aureus* and found that beef sausages had bacterial counts below 3 log 10 cfu/g [75]. The authors attributed this efficiency to good hygienic practice during the production process. Shin et al. [83] reported that there were no coliform bacteria detected in pork patties. Patarata et al. [71] noted that red wine in combination with garlic showed antibacterial activity against *C. sporogenes* and *Salmonella* in dry-cured sausages—chouriços. Similar results were obtained by Aquilani et al. [82]. They used grape seed and chestnut extract in the preparation of Cinta Senese dry-fermented sausage and found that the counts of *E. coli*, *L. monocytogenes*, *Clostridium* spp., *Salmonella* spp. and *Staphylococcus* spp. were low for up to 24 days of ripening. The authors attributed the efficiency of these extracts to the phenolic content. The addition of 1.56% winter savoy (*S. montana*) EO inhibited the spore formation of *C. perfingens* in mortadella-type sausage. [95]. Similar observations were made by Khaleghi et al. [70]. The authors noted that the TVC was particularly low for samples with the addition of 90 mg/kg barberry (*B. crataegina*) extract and nitrite (30 or 60 mg/kg). In addition, *C. botulinum* was not detected in samples during 30 days of storage, including in samples with no nitrite or plant extract added. Gyawali and Ibrahim [92] indicated that variations in the chemical and structural composition of phenolic compounds may result in differences in the antimicrobial properties of plant extracts. As Nowak et al. [87] reported, despite the high polyphenolic content of leaf extracts of *P. cerasus* and *R. nigrum*, their addition to the pork sausages did not stop the growth of psychotrophic and mesophilic bacteria after 28 days of storage. However, according to the literature, only *Pseudomonas* spp. were inhibited by leaf extracts during the entire storage period. Van Cuong and Thoa [77] found that the total count of bacteria and Enterobacteriaceae was lower in all samples with the addition of annatto seed powder, regardless of its quantity. However, the effect was similar to nitrite. The addition of extracts from *K. vitifolia* to dry-fermented sausage helped in decreasing the count of harmful microorganisms (e.g., *P. vulgaris*, *Bacillus subtilis* and *Aspergillus niger*). The strongest inhibitory effect was seen against *E. coli* [89].

### 5.4. Flavor and Smell

Nitrite is less known for its role in imparting a unique flavor or smell to cured meat products. During preparation, salt, spices and antioxidants are added to the meat. It is thought that if nitrite is added, it contributes to the creation of the characteristic cured flavor of the meat [64,65]. From the consumer point of view, the flavor of the meat is an important factor, in addition to the juiciness and tenderness of the product, thus determining its overall “eating quality” [96]. Table 5 presents data on various plant extracts and their effects on the flavor and smell of meat products. The application of high quantities of vegetable extracts, especially from celery, can result in a “vegetable taste” in the meat product. However, Riel et al. [67] prepared mortadella-type sausage with parsley extract added in various quantities (1.07, 2.14 and 4.29 g/kg). Sensory evaluation of their product showed that application of the highest amount of parsley extract resulted in products similar to the traditionally cured product, and did not create a vegetable taste. The authors also pointed out that parsley is less allergenic compared to celery extract. Furthermore, according to the panelists, samples with the addition of 2.14 and 4.29 g/kg powdered parsley extract were rated better than the sample prepared with 1.07 g/kg. The sensory evaluation revealed that the flavor of pork sausage prepared with paprika powder (0.04%) and blueberry powder (0.03%) was better than that of other sausages. In second place was ranked samples prepared with celery powder [85]. However, sensory analysis of sucuk with various amounts of beet extract (0.12%, 0.24% and 0.35%) did not show any difference in comparison to the control (150 mg/kg sodium nitrite) [84]. In addition, Shin et al. [83] did not find any difference between samples in terms of color, juiciness and tenderness. The authors stated that, with an increasing quantity of celery, the flavor of the product decreased. Spice extracts are well known for their flavor properties. In addition, *P. cerasus* and *R. nigrum* leaf extracts showed similar sensory qualities to those of traditional products [87]. Sueprasarn et al. [86] replaced nitrite with karanda extracts in Nham-fermented sausage. The application of 0.5% (*w/w*) of karanda extract proved optimal in the sensory evaluation. Kurćubić at al. [89] showed that samples prepared with the addition of *K. vitifolia* plant extract (12.5 g/kg) were rated similar to the control sample. However, according to Vossen et al. [80], the addition of dog rose extract to pork frankfurters did not create clear differences in texture parameters between samples. A similar observation was found by Çemtekin et al. [78]. Khaleghi et al. [70] reported that *B. crataegina* and nitrite were found to be less acceptable in comparison to samples prepared with 30 and 60 mg/kg extract and 90 mg/kg nitrite. In addition, cured (0.008% nitrite) pork sausage with adzuki bean extract (0.2%) presented a significantly lower score for odor and color parameters compared to the sausage without the extract. For the remainder of the parameters (flavor, taste and overall acceptance), the panelists did not find any difference [76]. Similar observations were reported by Aquilani et al. [82], where all three products were rated similarly. The complete replacement of nitrite by olive leaf extract (240 mg/100 g meat) in mortadella sausage resulted in an undesirable taste in the product [78]. Furthermore, panelists awarded chouriço made with the addition of red wine a good rating in comparison to samples prepared with only salt or with only nitrate. The combination of garlic and red wine resulted in a slightly brownish color of the product. Moreover, samples prepared with wine and in combination with other plant extracts exhibited an intense cured aroma [71]. These studies show that it is possible to produce meat products with plant extracts by replacing nitrite, and yet obtain the same flavor as that of traditional products. Hayes et al. [81] reported that tomato pulp powder imparts a sweet taste to the product. In addition, the effect of plant powder on the textural qualities of the product has also been studied. For example, the addition of 3% tomato pulp resulted in a negative effect, and decreased the texture and juiciness of the product. Moarefian et al. [90] obtained no differences in a sensory analysis of samples prepared with 20 and 40 ppm of EO of *M. piperita* combined with 100 and 80 ppm of sodium nitrite, respectively. Similar observations were obtained regarding the preparation of beef sausage with grape pomace. The authors obtained no differences in samples prepared with 1% or 2% pomace; however, in comparison to the control sample prepared with nitrite, grape pomace improved the odor and taste but reduced the color quality [74].

## 6. Conclusions

Nitrite is a multifunctional additive that is commonly used in the meat industry. However, due to the negative effect of nitrite on human health, it is important to reduce the amount of nitrite added to meat products. Plant extracts appear to be a suitable alternative for synthetic nitrite. In general, plant extracts show antioxidant and antimicrobial activities, can prevent the discoloration of meat products, and, in proper quantities, do not hamper the flavor of the product. It is notable that the incorporation of some plant extracts in combination with nitrite yields a synergistic effect (e.g., in the cases of beet, red grape pomace and green tea catechins), whereas some extracts (e.g., barberry and green tea) yield antagonistic effects. Therefore, it is important to study the nature of the extract in combination with nitrite. In other cases (e.g., red dragon fruit extract), a higher quantity of extract increases the antioxidant property of the product. In general, plant extracts decrease the lightness of the product, in addition to increasing its redness and yellowness. In most cases, the quantity of microorganisms present in a product prepared with a plant extract was reduced, or not detected at all. However, some authors suggest that this beneficial effect was not only an effect of the plant extract, but was also due to proper heat treatment and good hygienic conditions being in place during the preparation of the specific product (e.g., dry-fermented sausage). Moreover, in most cases, the addition of plant extract to meat products, in combination with a lower quantity (or complete absence) of nitrite, did not cause any negative effects on the products’ sensory qualities.

This review demonstrates that it is possible to use low quantities of nitrite in the preparation of meat products, and that the addition of plant extracts helps to achieve a product quality similar to that achieved using sodium nitrite alone. The major difficulty lies in choosing the optimal quantity of plant extract to apply. Therefore, further studies are still needed in this regard.

## Figures and Tables

**Table 1:** The general division of nitrate accumulation in vegetables.

Nitrate Content Expressed as mg/100 g Fresh Weight	Type of Vegetable
>2500	celery, cress, lettuce, spinach, rucola
from 1000 to <2500	Chinese cabbage, endive, leek, parsley
from 500 to <1000	turnip, savoy cabbage, cabbage
from 200 to <500	carrot, cucumber, pumpkin, broccoli
<200	potato, tomato, onion, eggplant, mushroom, asparagus

Table compiled according to [15].

**Table 2:** Application of plant extracts as a natural coloring agent in meat products with a reduced quantity of nitrite.

Meat Product	Storage Time [days]	Plant/Forms	Concentration	Nitrite Max. Amount (Control)/Nitrite Reduced Amount	Results	References
THERMAL TREATEMENT						
Mortadella type sausage	1, 7, 14, 21, 28	parsley/extract	1.07 g/kg, 2.14 g/kg, 4.29 g/kg	80 ppm/0 ppm	a* reduction during storage, b* higher for sample with higher amount of extract	[67]
beef mortadella	0, 1, 3, 6, 9, 12	olive oil/extract from leaves	240 mg/100 g, 240 mg/100 + nitrite (80, 60, 40, 20)	120 ppm/80 ppm, 60 ppm, 40 ppm, 20 ppm	synergistic effect of both additives on color	[88]
pork luncheon roll	2, 7, 14	tomato/pulp powder	0%, 1.5%, 3% + 0.0%, 0.05%, 0.1% nitrite addition	0.05%, 0.1%/0%	a* increase, L* reduction,	[81]
pork patties	0, 7, 14, 21, 28	pre-converted nitrite form Swiss Chard (*Beta vulgaris* var. cicla)and celery/powder	2%, 1% SC + 0.006 ppm nitrite	0.012%/0.006%	a*, b* parameters increase,	[83]
frankfurters	1, 20, 40, 60	dog rose (*R. canina)*/extract	180 g (extract from 5 g and 30 g of fruit)	0.1 g/kg/0 g/kg	influence on redness	[80]
ham slices	0, 3, 7, 10, 14, 21, 28, 35	celery juice/concentrate	75.6 g, 151.2	100 mg/kg,200 mg/kg/0 mg/kg	b* increase, L* decrease, a* similar to control	[68]
pork sausage	0, 14, 28	cherry (*Prunus cerasus* L.), blackcurrant (*Ribes nigrum* L.)/extracts from leaves	0.5 g/100 g, 1.0 g/100 g	1.8 g/100 g of meat/0 g	no influence on a* parameter, increase of b* parameter	[87]
pork sausage	over 4 weeks	celery, fruit, purple sweet potato, fruit+vegetable gardenia red, paprika + blueberry/powders, extract powders	0.8%, 0.6%, 0.45%, 0.5%, 0.04%, 0.07%	0.01%/no reduction	in general: positively influence on color	[85]
pork sausage	0,5 10, 15, 20	young radish, commercial vegetable (cabbage, *Brassica oleracea var. capitate* and Chinese cabbage, *Brassica rapa* L. spp. *pekinensis*)/ powder	250 g kg^−1^	0.087 g kg^−1^/0 g kg^−1^	a* stabilization	[69]
pork sausage	after production process	annatto (*Bixa Orellana* L.) seed (outer layer)	0.025%, 0.05%, 0.1%, 0.2%	150 ppm/37.5 ppm	increase redness, yellowness, decrease lightness	[77]
beef sausage cooked	2, 9, 16, 23, 30	barberry (*Berberis crataegina L*.) /extract	nitrite:extract [mg kg^−1^] 30:30, 30:60, 30:90, 60:90, 60:60, 60:90, 90:30, 90:60, 90:90	120 mg/kg ^−1^/90 mg/kg ^−1^, 60 mg/kg ^−1^, 30 mg/kg ^−1^	a* improvement	[70]
beef sausage	0, 10, 20, 30	red grape/ pomace	nitrite[ppm]: extract [%] 60:1.0030:1.060:2.0030:2.00	120 mg/kg/60 mg/kg, 30 mg/kg	L*, b* decrease	[72]
beef sausage	29	pomegranate peel, pistachio green hull/ extract	nitrite [ppm]:extract 100:250, 80:500, 60:750, 40:1000, 0:1250	120 ppm/100, 80, 60, 40, 0 ppm	color influence depending on amount and type of extracts	[74]
beef sausage	after production process	red dragon fruit peel (*Hylocereus polyrhizus*)/ extract	0%, 20%, 30%, 40%	no addition	a*, b* increase,	[75]
cooked turkey model system	0, 5, 10, 15, 30	guelder rose/powder (*Viburnum opulus* L.), hawthorn (*Crataegus monogyna*)/concentra-tes	1%, 5%, 10%	25 ppm, 50 ppm, 100 ppm, 156 ppm/0 ppm	a*, b* increased	[78]
NON-THERMAL TREATEMENT						
cured pork sausage, cooked sausage	1, 3, 5	Adzuki beans (*Vigna angularis*)/ extract	0.05%, 0.1%, 0.2%, 0.3%	0.008%/0%	a* increase	[76]
dry cured beef sausage	10, 20, 30	green tea (*Camellia sinensis* L.) catechins/ extract	nitrite:catechins [mg kg^−1^] 100:300, 125:300	75 mg/kg/100 mg/kg, 125 mg/kg	L* decline, synergistic values on b* parameter	[73]
pepperoni	0, 16, 46, 76	green tea/extract	0.05%, 0.05% and 0.009% nitrite	0.003%, 0.006%, 0.009%, 0.012%, 0.015%/no reduction	no strong color affection by extract addition	[79]
Chouriços cold dried, smoked sausages	7, 14, 30	red wine, red wine + garlic/----	7.5%, 7.5% + 1%	150 ppm/75 ppm	b* increase	[71]
Cinta Senese dry-fermented sausages	after 21	grape seed and olive pomace hydroxytyrosol, chestnut and olive pomace hydroxytyrosol/extract	10 g/kg	30 ppm/0 ppm	influence on a* and b*	[82]
sucuk fermented beef sausage	0, 56, 84	beetroot (*Beta vulgaris*)/powder	0.12%, 0.24%, 0.35%	150 mg/kg/100, 50, 0 mg/kg	a* increase, b*,L* decrease	[84]
Nham fermented pork sausage	0, 5, 10, 15, 20, 25, 30	Karanda (*Carissa carandas* Linn.)/extract	0.5% (*w/w*), 0.5% + 125 ppm nitrite	125 ppm/0 ppm	increase of b* and L* parameter	[86]

**Table 3:** Application of plant extracts as natural antioxidants in meat products with a reduced quantity of nitrite.

Meat Product	Storage Time [days]	Plant/Forms	Concentration	Nitrite Max. Amount (Control)/Nitrite Reduced Amount	Results	References
Thermal Treatement						
pork patties	0, 7, 14, 21, 28	pre-converted nitrite form Swiss Chard (*Beta vulgaris var. cicla*) and celery/powder	2%, 1% SC + 0.006 ppm nitrite	0.012%/0.006%	thiobarbituric acid reactive substances (TBARS) reduction	[83]
pork luncheon roll	2, 7, 14	tomato/pulp powder	0%, 1.5%, 3% + 0.0%, 0.05%, 0.1% nitrite addition	0.012%/0.006%	pro-oxidant effect of combination of both additives	[81]
frankfurters	1, 20, 40, 60	dog rose (*R. canina)/extract*	180 g (extract from 5 g and 30 g of fruit)	0.1 g/kg / 0 g/kg	antioxidant properties	[80]
pork sausage	0, 14, 28	cherry (*Prunus cerasus* L.), blackcurrant (*Ribes nigrum* L.)/ extracts from leaves	0.5 g/100 g, 1.0 g/100 g	1.8 g/100 g of meat/0 g	TBARS reduction	[87]
pork sausage	0,5 10, 15, 20	young radish, commercial vegetable (cabbage, *Brassica oleracea var. capitate* and Chinese cabbage, *Brassica rapa* L. spp. *pekinensis*)/powder	250 g kg^−1^	0.087 g kg^−1^/0 g kg^−1^	prevent lipid oxidation—similar to control	[69]
pork sausage	after production process	annatto (*Bixa Orellana* L.) seed (outer layer)/powder	0.025%, 0.05%, 0.1%, 0.2%	150 ppm/37.5 ppm	TBARS reduction, POV lower compared to control	[77]
pork sausage	----	celery, fruit, purple sweet potato, fruit+vegetable, gardenia red, paprika + blueberry/powders, extract powders	0.8%, 0.6%, 0.45%, 0.5%, 0.04%, 0.07%	0.01%/ no reduction	TBARS reduction	[85]
cooked sausage	2, 9, 16, 23, 30	*Mentha piperita/*essential oil	EO:nitrite [ppm] 20–100, 40–80, 60–60	120 ppm/100 ppm, 80 ppm, 60 ppm	TBARS significant reduction for sample with 20ppm of EO; slightly increase of PV value with higher amount of oil	[90]
beef sausage cooked	2, 9, 16, 23, 30	barberry (*Berberis crataegina L*.)/extract	nitrite:extract [mg kg^−1^] 30:30, 30:60, 30:90, 60:90, 60:60, 60:90, 90:30, 90:60, 90:90	120 mg/kg ^−1^/90 mg/kg ^−1^, 60 mg/kg ^−1^, 30 mg/kg ^−1^	potential antioxidant properties; negative interaction between nitrite and extract	[70]
beef sausage	0, 10, 20, 30	red grape /pomace	nitrite[ppm]:extract [%] 60:1.00 30:1.0 60:2.00 30:2.00	120 mg/kg/60 mg/kg, 30 mg/kg	synergistic properties against lipid oxidation process, possibly antioxidant properties of pomace (DPPH)	[72]
beef sausage	after production process	red dragon fruit peel (*Hylocereus polyrhizus*)/extract	0%, 20%, 30%, 40%	no addition	TBARS reduction, antioxidant properties increased along with extract amount	[75]
beef sausage	1, 8, 15, 22, 29	pomegranate peel, pistachio green hull/extract	nitrite: extract [ppm] 100:250, 80:500, 60:750, 40:1000, 0:1250	120 ppm/100, 80, 60, 40, 0 ppm	TBARS reduction, hydroperoxides reduction	[74]
chicken mince	0, 2, 4, 6	turmeric/powder	1000 ppm, 5000 ppm	200 ppm/0 ppm	TBARS reduction	[91]
cooked turkey model system	0, 5, 10, 15, 30	guelder rose (*Viburnum opulus* L.), hawthorn (*Crataegus monogyna*)/concentrates	1%, 5%, 10%	25 ppm, 50 ppm, 100 ppm, 156 ppm / 0 ppm	TBRAS reduction when 10% of additive	[78]
NON-THERMAL TREATEMENT						
cured pork sausage, cooked sausage	1, 3, 5	Adzuki beans (*Vigna angularis*)/extract	0.05%, 0.1%, 0.2%, 0.3%	0.008% / 0%	TBARS reduction	[76]
cured pork loins	after production process	fermented swiss chard/solution (in brine)	10%+32.2 ppm nitrite, 20% + 64.4 ppm, 30% + 96.6, 40% + 128.8	120 ppm/32.2 ppm, 64.4 ppm, 96.6 ppm, 128 ppm	TBARS reduction	[66]
Nham fermented pork sausage	0, 5, 10, 15, 20, 25, 30	Karanda (*Carissa carandas* Linn.)/extract	0.5% (*w/w*), 0.5% + 125 ppm nitrite	125 ppm / 0 ppm	combination with nitrite allows us to extract shelf-life	[86]
fermented dry sausage	20, 40, 60 (during storage)	*Kitaibelia vitifolia/* extract	3% *w/v* (30 g/kg), 10% *w/v* (12.5 g/kg)	27.5 g/kg / 0 g/kg	improvement antioxidant properties	[89]
sucuk fermented beef sausage	0, 28, 56, 84	beetroot (*Beta vulgaris*) /powder	0.12%, 0.24%, 0.35%	150 mg/kg/100, 50, 0 mg/kg	synergistic properties between nitrite and powder on TBARS reduction	[84]
dry cured beef sausage	10, 20, 30	green tea (*Camellia sinensis* L.) catechins /extract	nitrite:catechins [mg kg^−1^] 100:300,125:300	75 mg/kg/100 mg/kg, 125 mg/kg	synergistic antioxidant properties	[73]
pepperoni	0, 16, 46, 76	green tea /extract	0.05%, 0.05% and 0.009% nitrite	0.003%, 0.006%, 0.009%, 0.012%, 0.015%/no reduction	TBARS reduction, prooxidant properties in combination with nitrite	[79]

**Table 4:** Application of plant extracts as natural antimicrobials in meat products with a reduced quantity of nitrite.

Meat Product	Storage Time	Plant/Forms	Concentration	Nitrite Max. Amount (Control)/Nitrite Reduced Amount	Results	References
THERMAL TREATEMENT						
mortadella type sausage	1, 7, 14, 21, 28	parsley/extract	1.07 g/kg, 2.14 g/kg, 4.29 g/kg	80 ppm/0 ppm	*L. monocytogenes* reduction (depending on amount of extract addition)	[67]
pork patties	0, 7, 14, 21, 28	pre-converted nitrite form Swiss Chard (*Beta vulgaris var. cicla*) and celery /powder	2%, 1% SC + 0.006 ppm nitrite	0.012%/0.006%	no bacteria present during storage (*E. coli*, coliform bacteria)	[83]
ham slices	0, 3, 7, 10, 14, 21, 28, 35	celery juice/concentrate	75.6 g, 151.2	100 mg/kg, 200 mg/kg/0 mg/kg	decrease *L. monocytogenes*	[68]
pork sausage	12, 24 h	young radish, commercial vegetable (cabbage, *Brassica oleracea var. capitate* and Chinese cabbage, *Brassica rapa* L. spp. *pekinensis*)/powder	250 g kg^−1^	0.087 g kg^−1^/0 g kg^−1^	antibacterial properties of young radish against *L. monocytogenes, S. aureus*	[69]
pork sausage	after production process	annatto (*Bixa Orellana* L.) seed (outer layer) /powder	0.025%, 0.05%, 0.1%, 0.2%	150 ppm/37.5 ppm	no *E. coli* detected, TPC and VRB similar to the control	[77]
pork sausage	14, 28	cherry (*Prunus cerasus* L.), blackcurrant (*Ribes nigrum* L.)/extracts from leaves	0.5 g/100 g, 1.0 g/100 g	1.8 g/100 g of meat/0 g	strong antimicrobial activity against *Pseudomonas*	[87]
meat (pork) model system	0, 3, 6, 9, 12	cherry powder and lemon powder; green tea extract and lime powder; cranberry powder and grape seed extract; cherry powder, lemon powder, cranberry powder, VegStable /extracts, powders	150 ppm of nitrite + 0.6% and 60 ppm; 150 ppm nitrite + 1000 ppm and_60 ppm;150 ppm of nitrite + 1% or 2%, or 3% + 0.5%; 50 ppm of nitrite + 0.6% + 60 ppm + 1% + 0.7%	200 ppm/150 ppm, 50 ppm, in combination	inhibition properties against *L. monocytogenes*	[93]
beef fillet	during 30 days of storage	*Zataria multiflora Boiss Satureja bachtiarica Bunge, Origanum vulgare L.* /essential oils	0.355%, 0.71% v.w-1, 0.275%, 0.55% v.w-1, 0.395%, 0.79% v.w-1	200 mg kg^−1^, 100 mg kg^−1^, 0 mg kg^−1^ in combination with exctarc	inhibition properties against *C. perfringens, C. sporogenes*	[94]
mortadella type sausage	1, 10, 20, 30	winter savory (*Satureja montana* L.) /essential oil	0.0%, 0.78%, 1.56%, 3.125%, + 100 or 200 ppm nitrite addition	200 mg/kg, 100 mg/kg, 0 mg/kg	synergistic effect between both additives for *L. monocytogenes* inhibition	[95]
beef sausage	after production process	red dragon fruit peel (*Hylocereus polyrhizus*) /extract	0%, 20%, 30%, 40%	no addition	antimicrobial properties of extract in vitro against *E. coli, Salmonella, S. enterica, S. aureus*	[75]
beef sausage cooked	2, 9, 16, 23, 30	barberry (*Berberis crataegina L*.) /extract	nitrite:extract [mg kg^−1^] 30:30, 30:60, 30:90, 60:90, 60:60, 60:90, 90:30, 90:60, 90:90	120 mg/kg^−1^/90 mg/kg^−1^, 60 mg/kg^−1^, 30 mg/kg^−1^	at the end of storage TVC on stable level, similar to control, no *C. perfringens* presence	[70]
NON-THERMAL TREATEMENT						
*Chouriços* cold dried, smoked sausages	7, 14, 30	red wine, red wine + garlic/----	7.5%, 7.5% + 1%	150 ppm/75 ppm	inhibitory properties against *Salmonella*; no influence of additives on *Cl. sporogenes*	[71]
*Cinta Senese* dry-fermented pork sausages	after 21 days	grape seed and olive pomace hydroxytyrosol, chestnut and olive pomace hydroxytyrosol/extract	10 g/kg	30 ppm/0 ppm	*L. monocytogenes, Salmonella, Cl. botulinum* - absent/below the limit of required	[82]

**Table 5:** Application of plant extracts as natural compounds affecting the taste and smell of meat products with a reduced quantity of nitrite.

Meat Product	Plant/Forms	Flavor	Odor	Impact/Effect	References
THERMAL TREATEMENT					
Morta della type sausage	parsley extract	no differences between samples or and between samples and control	similar to control	acceptance product with 2.14 and 4.29 g/kg of extract	[67]
beef morta della	olive oil/extract from leaves	decrease along with decreased amount of nitrite addition; bitter taste appeared	---------	combination of oil (240mg/100 g) and nitrite (80 and 60 ppm) is optimal	[88]
pork patties	pre-converted nitrite form Swiss Chard (*Beta vulgaris var. cicla*) and celery /powder	negative influence of celery powder on taste	--------	addition of 1% of swiss chard had higher overall acceptability	[83]
pork luncheon roll	tomato/pulp powder	higher pulp concentration increased the sweetness in product and decrease the juiciness	no effect on cured aroma	addition of 1.5% of pulp powder is an optimal amount	[81]
frankfurters	dog rose	---------	--------	addition of extract from 30 g of fruit increase hardness of product	[80]
pork sausage	celery, fruit, purple sweet potato, fruit+vegetable, gardenia red, paprika+blueberry / powders, extract powders	samples with fruit, purple sweet potato, fruit+vegetable/powders, extract powders addition rated very low	samples with fruit, purple sweet potato, fruit + vegetable/powders, extract powders	very good rating of samples with celery and paprika + blueberry	[85]
pork sausage	cherry (*Prunus cerasus* L.), blackcurrant (*Ribes nigrum* L.)/extracts from leaves	taste similar to sample with nitrite	odor similar to sample with nitrite	no negative effects of extract addition on product—samples with extract similar to the control sample	[87]
cooked sausage	*Mentha piperita*/essential oil	no significant differences in relation to control	no significant differences in relation to control	no significant differences in odor and taste between samples	[90]
beef sausage cooked	barberry (*Berberis crataegina L*.)/extract	samples with 30:90 and 60:90 extract addition rated better than control and 90:90	no differences between all samples	addition of 30:90 and 60:90 mg kg^−1^ extract/nitrite is optimal	[70]
beef sausage	red grape/pomace	taste, texture improvement (regardless the concentration)	no differences between samples with extract and control	color deterioration along with higher amount of pomace; addition of extract in amount of >1% decrease overall acceptability	[72]
beef sausage	pomegranate peel, pistachio green hull/extract	higher amount of extract may decrease taste, reduction of nitrite with simultaneous extract addition did not cause any noticeable differences in taste, compared to control	higher amount of extract may decrease odor	possible to produce sausage with nitrite reduced to 60 ppm and extract addition	[74]
cooked turkey model system	guelder rose (*Viburnum opulus* L.), hawthorn (*Crataegus monogyna*)/concentrates	--------	--------	no differences between samples in texture profile	[78]
NON-THERMAL TREATEMENT					
cured pork sausage	Adzuki beans (*Vigna angularis*)/extract	no difference between products with extract addition for taste, overall acceptance	lower score for odor and color	extract addition may cause change of color and odor	[76]
fermented dry sausage	*Kitaibelia vitifolia*/extract	sample with addition of 3% (*w/v*) extract has been scored lower	sample with addition of 3% (*w/v*) extract has been scored lower	product with extract (10% *w/v*) similar to the control with nitrite	[89]
fermented pork sausage Nham	karanda (*Carissa carandas* Linn.) / extract	no differences in relation to the control	no differences in relation to the control	product with 0.25% and 0.5% of extract addition similar to control with nitrite addition	[86]
fermented beef sausage sucuk	beetroot (*Beta vulgaris*)/powder	--------	--------	product with extract (all ratios) similar to the control with nitrite addition	[84]
*Cinta Senese* dry-fermented pork sausages	grape seed and olive pomace hydroxytyrosol, chestnut and olive pomace hydroxytyrosol/extract	no differences in relation to the control	no differences in relation to the control	no negative influence of extract on product; changes found with regard to color	[82]
*Chouriços* cold dried, smoked sausages	red wine, red wine + garlic/-----	-------	aroma similar between all samples	combination with garlic has a negative influence on color, wine combined with garlic gives positive results in the context of product purchase	[71]
